# Fatal diving: could it be an immersion pulmonary edema? Case report

**DOI:** 10.1007/s00414-022-02809-x

**Published:** 2022-03-14

**Authors:** France Evain, Pierre Louge, Rodrigue Pignel, Tony Fracasso, Frédéric Rouyer

**Affiliations:** 1 Forensic Pathology, University Center of Legal Medicine, Geneva University Hospitals and University of Geneva, rue Michel-Servet 1, CH-1211 Geneva 4, Switzerland; 2 Acute Medicine Department, Hyperbaric Medicine Unit, Geneva University Hospitals, rue Gabrielle-Perret-Gentil 4, 1205 Geneva, Switzerland; 3 Critical Care Unit of the Emergency Division, Acute Medicine Department, Geneva University Hospitals, rue Gabrielle-Perret-Gentil 4, 1205 Geneva, Switzerland

**Keywords:** Diving, Pulmonary, Edema, Autopsy, Case report

## Abstract

Immersion pulmonary edema is a rare, underrecognized, and potentially lethal pathology developing during scuba diving and other immersion-related activities (swimming or apnoea). Physiopathology is complex and not fully understood, but its mechanisms involve an alteration of the alveolo-capillary barrier caused by transcapillary pressure elevation during immersion, leading to an accumulation of fluid and blood in the alveolar space. Diagnosis remains a challenge for clinicians and forensic practionner. The symptoms begin during ascent, with cough, frothy sputum, and hemoptysis. Auscultation reveals signs of pulmonary edema. Pulmonary CT scan, which is the radiological exam of choice, shows ground glass opacities and interlobular thickening, eventually demonstrating a patterned distribution, likely in the anterior segments of both lungs. Apart from the support of vital functions, there is no specific treatment and hyperbaric oxygen therapy is not systematically recommended. We present a case of fatal IPE occurring in a recreational diver who unfortunately died shortly after his last dive. Diagnosis was made after complete forensic investigations including post-mortem-computed tomography, complete forensic autopsy, histological examination, and toxicological analysis.

## Introduction

Most diving injuries and deaths are related to decompression injuries. Immersion pulmonary edema (IPE) was first described in 1989 [1]. This rare and acute pathology develops during scuba diving or other immersion-related activities (swimming and apnoea) [2, 3], even in individuals in general good health [2, 4], but more often in males over 50 years of age [5, 6]. The symptoms begin during ascent, with cough, frothy sputum, and hemoptysis [5]. Pulmonary CT scan shows ground glass opacities and interlobular thickening, eventually demonstrating a patterned distribution [5–7], likely in the anterior segments of both lungs. The occurrence of IPE is favored by cold water, hypertension, strenuous exercise, and thigh wetsuits. Its incidence is estimated at 1.1% of all diving accidents [3, 7–10]. However, IPE is largely underdiagnosed, since it can remain asymptomatic, and is often underrecognized by medical and forensic practitioners [6]. The recurrence rate is estimated at 30%, although former divers may have stopped this activity after the first episode [3, 5, 8, 11]. Symptoms often develop within 10 min after immersion and worsen during ascent [6, 12]. Patients classically experience dyspnoea, frothy sputum, and/or haemoptysis, with possible dizziness, loss of consciousness, and even death [3, 4, 7, 8, 10].

## Case presentation

One morning in late December, a 56-year-old man in generally good health was diving in Geneva Lake with an experienced diver using air open-circuit and was dressed with a neoprene wet diving suit. He was a recreational diver who started this activity 6 months prior. At this time of year, the water temperature was rather cold (mean temperature during the dive, 7 °C).

After 10 min at 19.5 m under the surface, he started to cough and had difficulty to breath. Both divers started an ascent, without violation of the decompression procedure and keeping the regulator in their mouths. When he arrived at the surface, he was spitting up blood. The total diving time was 13 min. Back on the boat, he lost consciousness and had cardiac arrest.

A rescuer present on the boat immediately performed basic life support (BLS) (no-flow = 0 min). The patient arrived on the beach 5 min later undergoing BLS, and a resuscitation team with an emergency physician immediately started advanced cardiac life support (ACLS).

The first cardiac rhythm identified was non-shockable. Cardiopulmonary resuscitation (CPR) was conducted according to guidelines. The patient presented with an alternating shockable and non-shockable rhythms and was intubated. There was bloody foam in the oropharynx. The patient was transported by helicopter to Geneva University Hospitals, which is the only hospital in the area equipped with a hyperbaric chamber. Advanced CPR was continued during the flight. On admission to the emergency department, low-flow was estimated at 84 min. Pulseless electrical activity (PEA) alternating with ventricular fibrillation episodes requiring two electric shocks was recorded. No cardiac mechanical activity was identified during a focused cardiac ultrasound performed during resuscitation. Arterial blood gas analysis showed severe metabolic acidosis (pH = 6.38 adjusted to temperature, normal 7.35–7.45) with hyperlactatemia (27 mmol/L, normal: 0.5–1.6 mmol/L). After multidisciplinary discussion, the option of implanting an ECMO was not chosen. Death was pronounced 20 min after his admission to the hospital and 104 min of unsuccessful CPR.

**Fig. 1:**
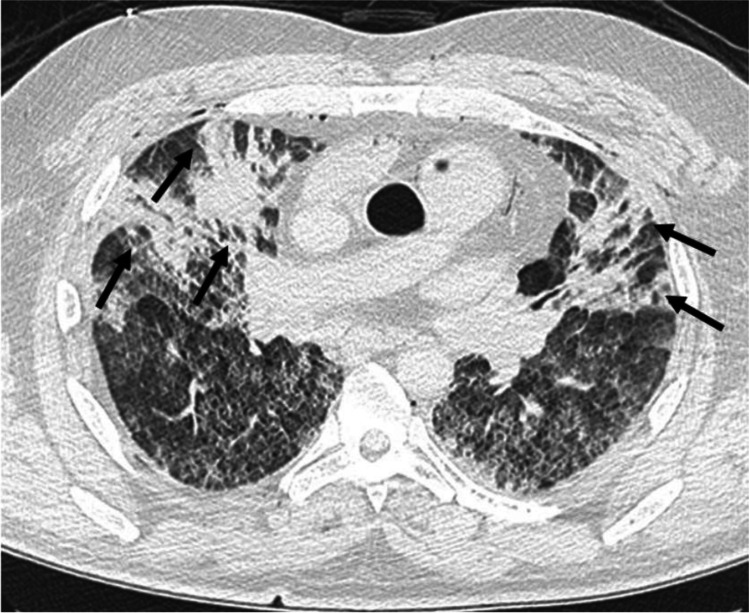
Post-mortem computed tomography (PMCT) showing diffuse bilateral interstitial thickening and panlobar air space consolidations in anterior segments of each lobes

A medicolegal autopsy was performed the day after. According to police investigations, the diving equipment did not show any dysfunction. Post-mortem computed tomography (PMCT) revealed diffuse bilateral interstitial thickening, panlobar air space consolidations in the anterior segments of each lobe (Fig. 1), and pulmonary emphysema.

**Fig. 2:**
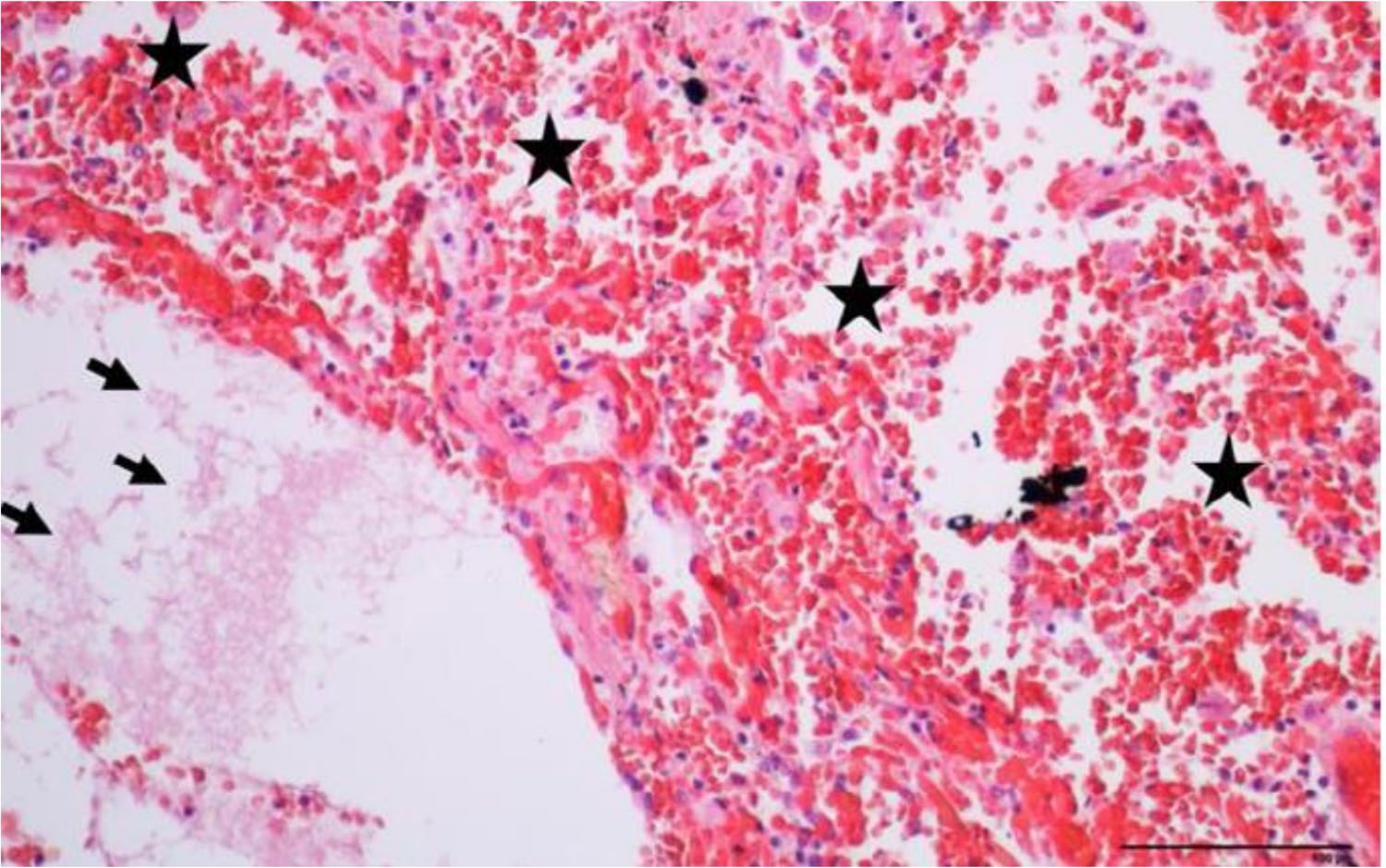
Upper left lobe lung parenchyma showing intra-alveolar hemorrhage (star) with fluid in alveolar spaces (arrow) (hematoxylin and eosin, × 200)

Gross autopsy findings included mild obesity (body mass index of 33 kg/m^2^), small amount of foam and blood in the distal airways, heavy and firm lungs (940 g right and 820 g left), global cardiac hypertrophy (540 g, normalized for BMI), fatty streaks at the pulmonary and coronary arteries, and nonspecific stress/agonal-related findings (subpleural petechiae, pinpoint hemorrhages of gastric mucosa). Neither typical signs of drowning (such as emphysema aquosum or subpleural hemorrhages known as Paltauf spots) nor fresh myocardial damage were found at gross observation and microscopic examination. The histology of the lungs showed intra-alveolar hemorrhage, fluid in the alveolar spaces (Fig. 2), and a few hemosiderophages. Post-mortem biochemistry revealed a normal NT-proBNP level at 132 ng/L (normal < 900 ng/L for patients 50 to 75 years old) and elevated level of hs-troponin T (1319 ng/L, normal < 14 ng/L) due to hemolysis of the sample. Toxicological analyses were negative.

## Discussion

In many cases of diving fatalities, IPE initiating cardiac decompensation or loss of consciousness in water is suspected. As a result, it is difficult to interpret eventual drowning-related findings at autopsy. In our case, the diving device remained in the mouth of the victim and the initial loss of consciousness occurred on the support boat.

Lung lesions found at autopsy could also be attributed to CPR, but their location and type are not typical of those usually found [13, 14]. Post-mortem examination revealed no typical signs of drowning. Therefore, the lesions observed at autopsy and virtopsy can only be secondary to IPE.

IPE is an underrecognized pathology in swimming and scuba diving activities. Confusion with other scuba diving pathologies is frequent but the treatment of IPE, when isolated, does not require systematic hyperbaric oxygen therapy.

IPE stems from anatomical and functional alterations of the alveolo-capillary barrier secondary to elevation of transcapillary pressure following immersion, with subsequent capillary damage (capillary stress failure) and transudation of fluid and blood into the alveolar space [6]. Several mechanisms are proposed:

- Immersion increases hydrostatic pressure on the body, leading to central redistribution of peripheral circulation (blood pooling) with elevation of pulmonary circulation and cardiac output [15]. Intensification of ventricular preload and afterload, as well as central blood pooling, leads to drastic elevation of the pulmonary capillary pressure and capillary stress failure [5, 16], with leakage of fluid and blood into the alveolar space [11, 17]. This phenomenon can eventually be intensified by exposition to cold water (peripheral vasoconstriction) [7], hypertension, cardiac hypertrophy, emotional stress, thigh diving suit, overhydration [18], and strenuous exercise [5].
- Hydrostatic pressure with central blood pooling following immersion causes an increase of work in the right ventricle, a mismatch of output volume between both ventricles [7, 14, 16], and an increase of pulmonary capillary pressure. The latter results in capillary stress failure and edema formation. Left ventricular dysfunction and valvular disease [4, 16] may foster mismatch of output volume and occurrence of IPE.
- Elevation of pulmonary blood pressure and ambient hydrostatic pressure during immersion lessens lung compliance [6]. Moreover, the air inhaled from the diving cylinder is dense and its flowing resistance is high [5]. These elements result in an enhancement of respiratory work with diminution of alveolar pressure during inspiration, stretching of alveolar walls [5], and an increase of transcapillary pressure, leading to capillary damage [3, 15]. A maladjusted regulator (inducing further airflow resistance), emotional stress (associated with hyperpnoea and/or jerky breathing), and exercise may enhance respiratory work and foster capillary damage [5].

Symptoms of IPE are known to worsen during the ascent [6, 12]. This can be explained by the natural evolution of the disease, damage of pulmonary capillaries due to filtration of air bubbles during decompression (according to Henry’s law), and expansion of intrathoracic gas (according to Boyle’s law) with redistribution of pulmonary edema. Moreover, the drop of alveolar pressure further increases the already elevated alveolo-capillary pressure gradient, worsening capillary damage [6, 12].

The diagnosis relies on clinical history (beginning of symptoms during the dive and worsening during ascent, cough, frothy sputum, and haemoptysis) [5]. Furthermore, pulmonary CT scan shows ground glass opacities, dilatation of pulmonary veins, interlobular thickening, and/or pleural effusion [6]. Ground glass opacities and interlobular thickening may demonstrate a patterned distribution [5–7], likely in the anterior segments of both lungs as in the case described above. This can be explained by the manifestation of pulmonary lesions in a sloping position (areas of greater transmural pressure during the dive) combined with the horizontal position of the diver in the water [6]. Blood testing (whether ante- or post-mortem) includes cardiac biomarkers (troponins, BNP), which are usually elevated (as opposed to decompression sickness) [3, 10]. However, normal levels of these biomarkers do not exclude the diagnosis, since their elevation takes several hours [2, 3].

The first measure is to remove the victim from the water to release the effects of hydrostatic pressure, removal of the wetsuit, and warming up to lessen central blood pooling [19]. Decompression stops should be respected if possible but should not delay the diver’s ascent and exit from the water. If no life-threatening symptom is suspected, the victim should be placed in the sitting position and receive highly concentrated oxygen with a facial mask before being transported to a hospital with an intensive care unit and a hyperbaric chamber. Invasive or non-invasive ventilation (NIV) with continuous positive airway pressure can be useful to ensure correct oxygenation and treat pulmonary edema. Hyperbaric oxygen therapy is indicated only in case of associated decompression sickness. A continuous infusion of nitroglycerin could be indicated in order to reduce right ventricular preload and left ventricular afterload. Diuretics are not systematically indicated due to the frequent dehydration associated with swimming and scuba diving activities. Inhaled beta 2 agonist drugs could be an alternative favoring water alveolar clearance. More data is required to collect proof of efficacy and standardize the treatment of IPE. In most cases, the patient remains in the hospital for 2–3 days and is banned from diving and other swimming activities for 1 month. Further investigations must be carried out to look for an unknown comorbidity, such as hypertension, cardiomyopathy, diabetes, or dyslipidaemia. Evaluation of respiratory function is recommended [20]. Fostering factors should be avoided (dive in temperate water, well-fitted wetsuit, set regulator, limitation in depth and duration of diving, and avoidance of overhydration and hyperoxia).

## Conclusion

IPE is a rare and underestimated pathology in scuba diving and swimming activities, but it can lead to a fatal issue. Post-mortem diagnosis of IPE is challenging. It rests on police investigation (technical expertise in order to exclude malfunction of diving devices, diving profile analysis, audition of witnesses, water temperature), access to medical information (classical cough, frothy sputum and haemoptysis, worsening during ascent), complete medicolegal autopsy (including histological examination, notably of the lungs) to exclude alternative cause of death, signs of drowning and assess comorbid conditions, and toxicological analysis. PMCT is an important diagnosis tool and shows characteristic ground glass opacities and interlobular thickening of the lungs, often demonstrating a distribution in the anterior segments of both lungs.
